# Vogt-Koyanagi-Harada Syndrome following COVID-19 and ChAdOx1 nCoV-19 (AZD1222) vaccine

**DOI:** 10.1186/s40942-021-00319-3

**Published:** 2021-08-30

**Authors:** Janaína Jamile Ferreira Saraceno, Guilherme Macedo Souza, Luciana Peixoto dos Santos Finamor, Heloisa Moraes Nascimento, Rubens Belfort

**Affiliations:** 1 Department of Ophthalmology, Hospital Sao Rafael, Salvador, Brazil; 2 Department of Ophthalmology and Visual Science, Federal University of Sao Paulo, Sao Paulo, Brazil; 3 Research Department, Instituto Paulista de Estudos e Pesquisas em Oftalmologia/Instituto da Visão (IPEPO), Sao Paulo, Brazil

## Abstract

The challenge of COVID-19 has rapidly changed medical management worldwide. The relatively small time from pandemic to vaccines regulatory approval triggered a race toward vaccines development. However, important questions regarding SARS-CoV-2 vaccines remain. A case of complete Vogt-Koyanagi-Harada (VKH) Syndrome that occurred 4 days following SARS-CoV-2 vaccination and another patient that developed VKH 14 days post COVID-19 clinical onset are presented. A causal relationship between COVID-19 and uveitis may exist.

## Introduction

Vogt-Koyanagi-Harada (VKH) syndrome is a multisystem disorder with chronic, bilateral, non-necrotizing, granulomatous panuveitis and exudative retinal detachment [1–5]. The etiology of VKH remains unknown, and the pathogenesis is thought to be multifactorial and primarily targets the choroidal layer. Many etiologies were previously reported, most of which were immunogenetic and environmental. It is a T-cell-mediated autoimmune response against one or more antigenic components of melanocytes [6–8].

Previous reports demonstrate VKH association with interferon-alpha, pembrolizumab and dabrafenib/trametinib therapy, as well as after vaccination for influenza, yellow fever and BCG. Immunological mechanisms and dysregulation of the immune system may play a significant role in the association between VKH disease and COVID-19 [9–15].

Rare descriptions of post-COVID-19 immune-mediated conditions such as uveitis, Guillain-Barré syndrome or systemic lupus erythematosus have been published. It is speculated that SARS-CoV-2 can disturb self-tolerance and trigger autoimmune responses through cross-reactivity with host cells [16–18].

A case of complete VKH that occurred 4 days following vaccination with ChAdOx1 nCoV-19 (AZD1222) and another patient that developed VKH 14 days post COVID-19 clinical onset are presented. To our knowledge, no previous reports have indicated that SARS-CoV-2 infection or the vaccine may be related to the VKH.

## Cases reports

### Patient 1

A 62-year-old healthy female patient developed a severe headache and tinnitus 2 days after receiving COVID-19 immunization with the Oxford-AstraZeneca Chimpanzee Adenovirus Vectored Vaccine ChAdOx1 nCoV-19 (AZD1222). Two days later she developed an acute loss of vision in both eyes.

**Fig. 1:**
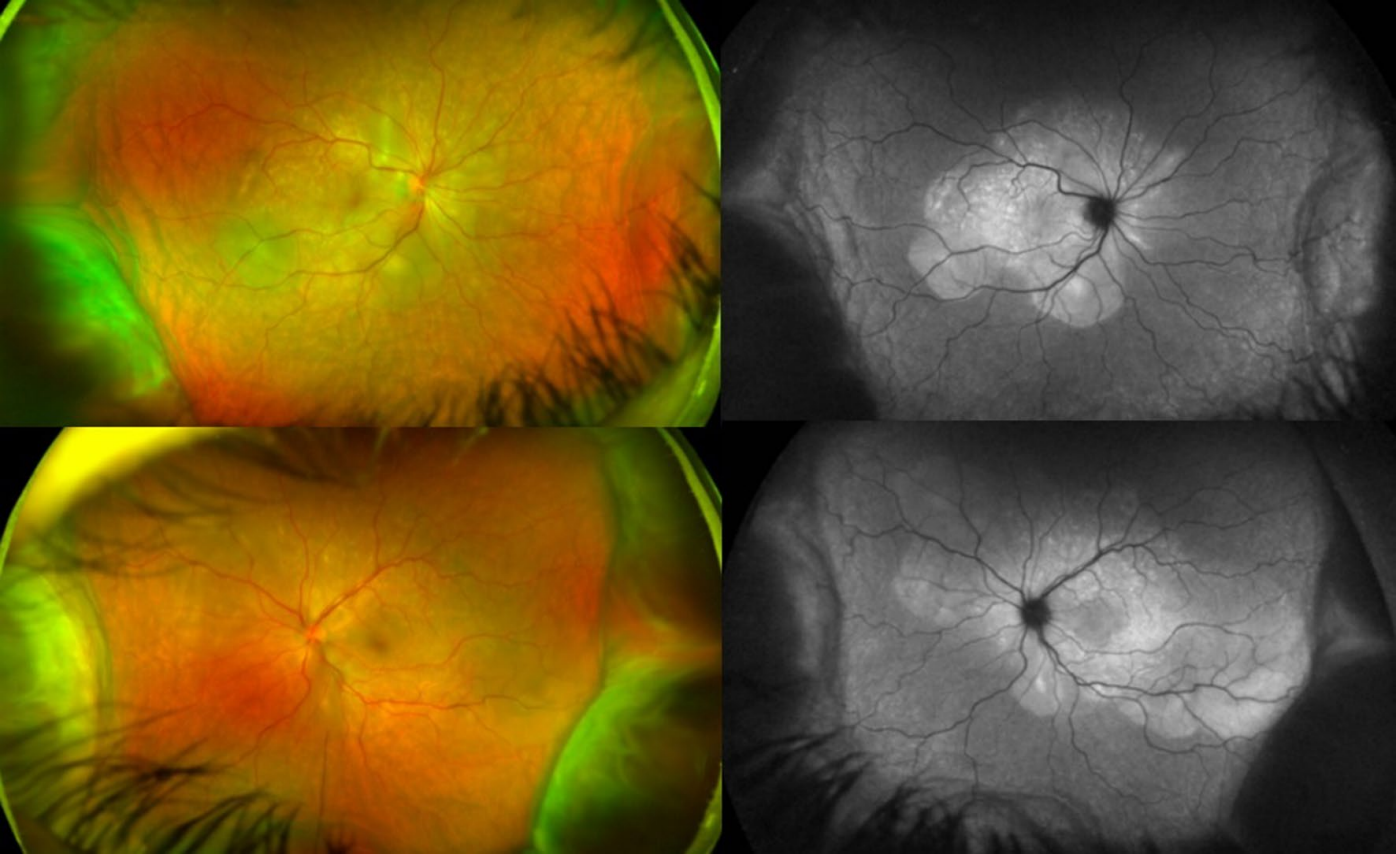
(Patient 1) Fundoscopy and autofluorescence of both eyes showing serous retinal detachment and optic disc hyperemia

**Fig. 2:**
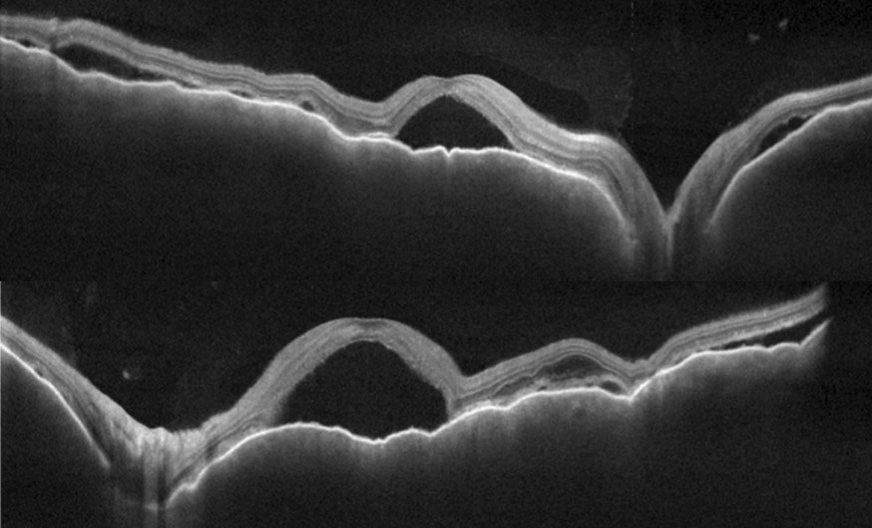
(Patient 1) OCT of both eyes showing serous retinal detachment with bacillary layer detachment

BCVA was 20/600 in OD and 20/200 in OS, IOP was 14 mmHg OU and the slit-lamp examination showed a mild inflammation in the anterior chamber with 2+ cells and 1+ of vitreous cells OU. Fundus examination showed a serous retinal detachment and optic disc hyperemia OU (Fig. 1). OCT showed bilateral serous retinal detachment, bacillary layer detachment and subretinal hyperreflective dots (Fig. 2).

### Patient 2

A 37-year-old female patient with a 15 days history of blurred vision with metamorphopsia in both eyes, associated with tinnitus and no prior history of eye trauma or intraocular surgery was seen. Two weeks before she had experienced headache, anosmia and fever and tested positive for the SARS COV-2 on rt-PCR test.

**Fig. 3:**
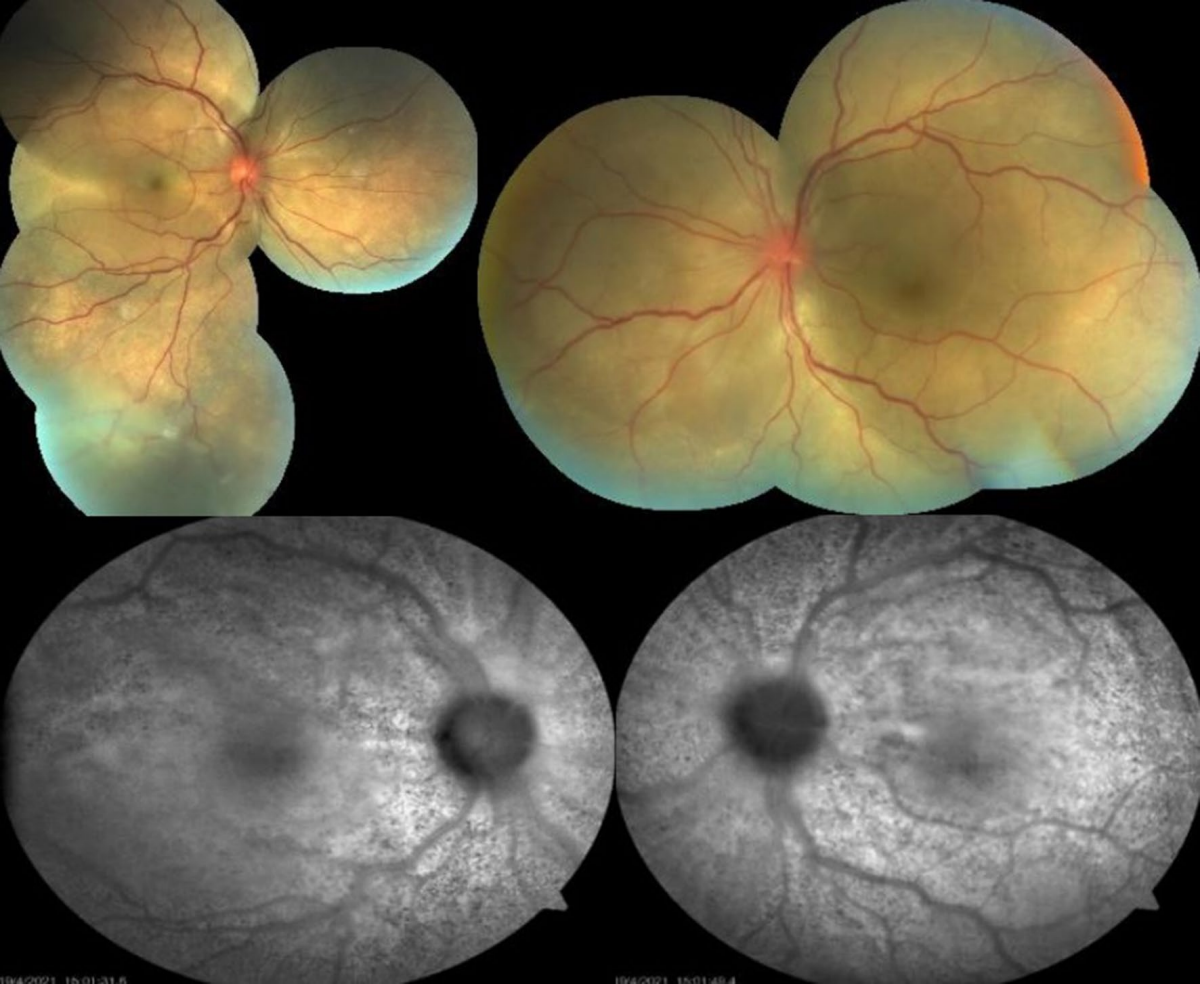
(Patient 2) Fundoscopy and autofluorescence of both eyes showing serous retinal detachment, optic disc hyperemia and choroidal inflammation

**Fig. 4:**
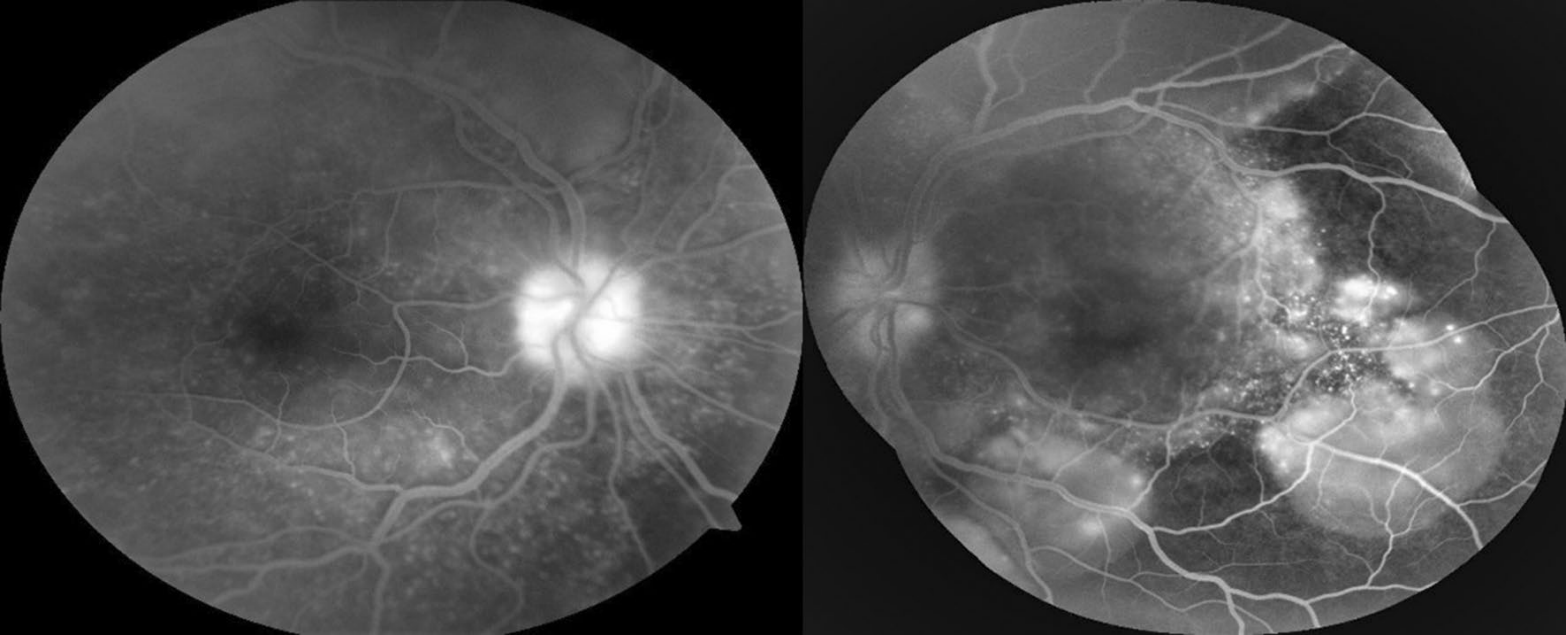
(Patient 2) Fluorescein angiography of both eyes

**Fig. 5:**
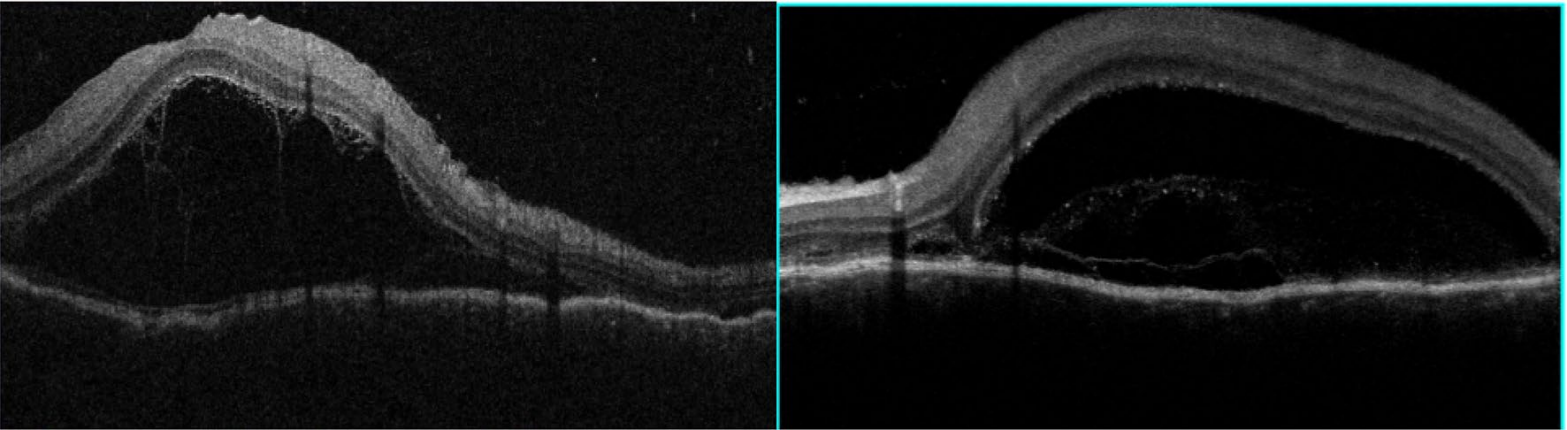
(Patient 2) OCT of both eyes showing serous retinal detachment with bacillary layer detachment

BCVA was hand motion in OU and intraocular pressure was normal. Slit-lamp examination showed granulomatous keratic precipitates (KP) in OD, and mild vitritis OU. Fundus examination showed serous retinal detachment with an inferior bullous detachment and optic disk hyperemia in OU (Fig. 3), fluorescein angiography revealed bilateral optic disk hyperfluorescence due to leakage and multiple hyperfluorescence pinpoints (Fig. 4), OCT revealed a bilateral serous retinal detachment with bacillary detachment (Fig. 5), characterizing initial onset.

In both cases, systemic evaluation was nonspecific with mononuclear cells in the cerebrospinal fluid and infectious work-up was negative. Both were diagnosed as complete VKH syndrome, according to the Revised Diagnostic Criteria (RDC) [3].

**Fig. 6:**
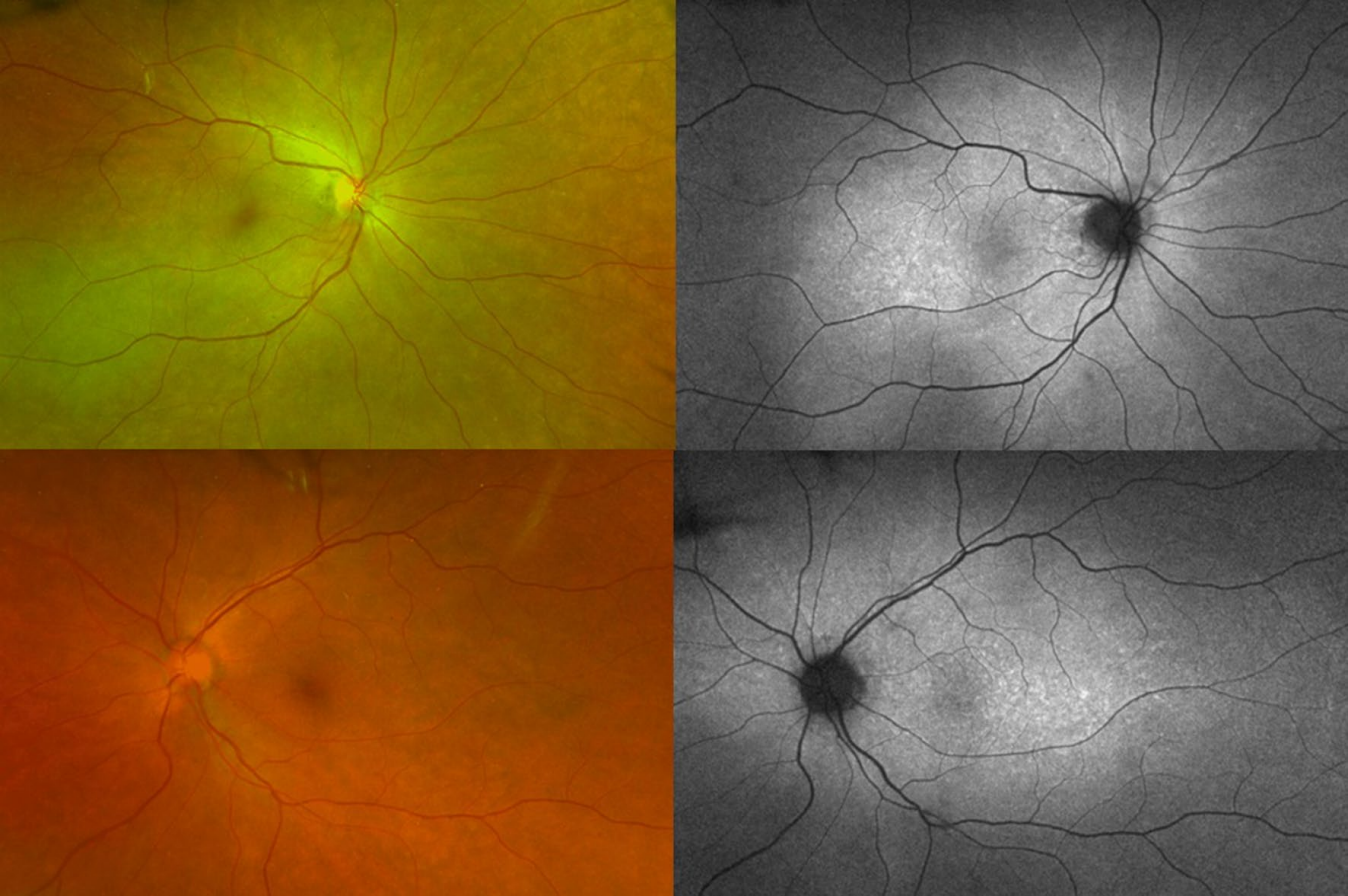
(Patient 1) Fundoscopy and autofluorescence of both eyes showing improvement of the serous retinal detachment

**Fig. 7:**
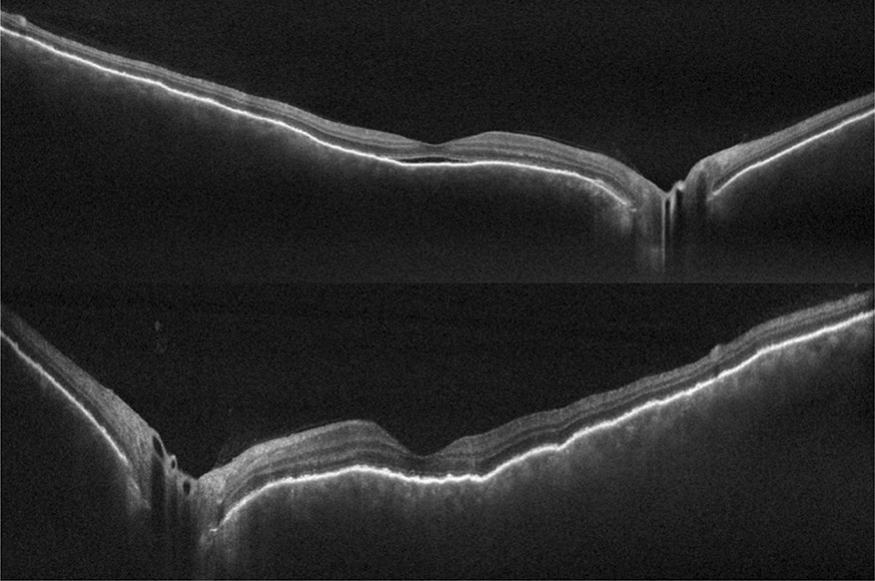
(Patient 1) OCT of both eyes showing improvement of the serous retinal detachment and bacillary layer detachment

Patient 1 was treated with oral systemic prednisone (1.5 mg/kg/day)—intravenous therapy was avoided due to the severity of the pandemic at that time and the restriction of available hospital beds—and within 4 days the visual acuity improved to 20/60 OD and 20/80 OS. She continued to be followed up, using regressive oral corticosteroid therapy, and after 3 weeks, evolved with BCVA of 20/20 OU, no signs of inflammatory activity and disappearance of the retinal detachment (Figs. 6 and 7).

**Fig. 8:**
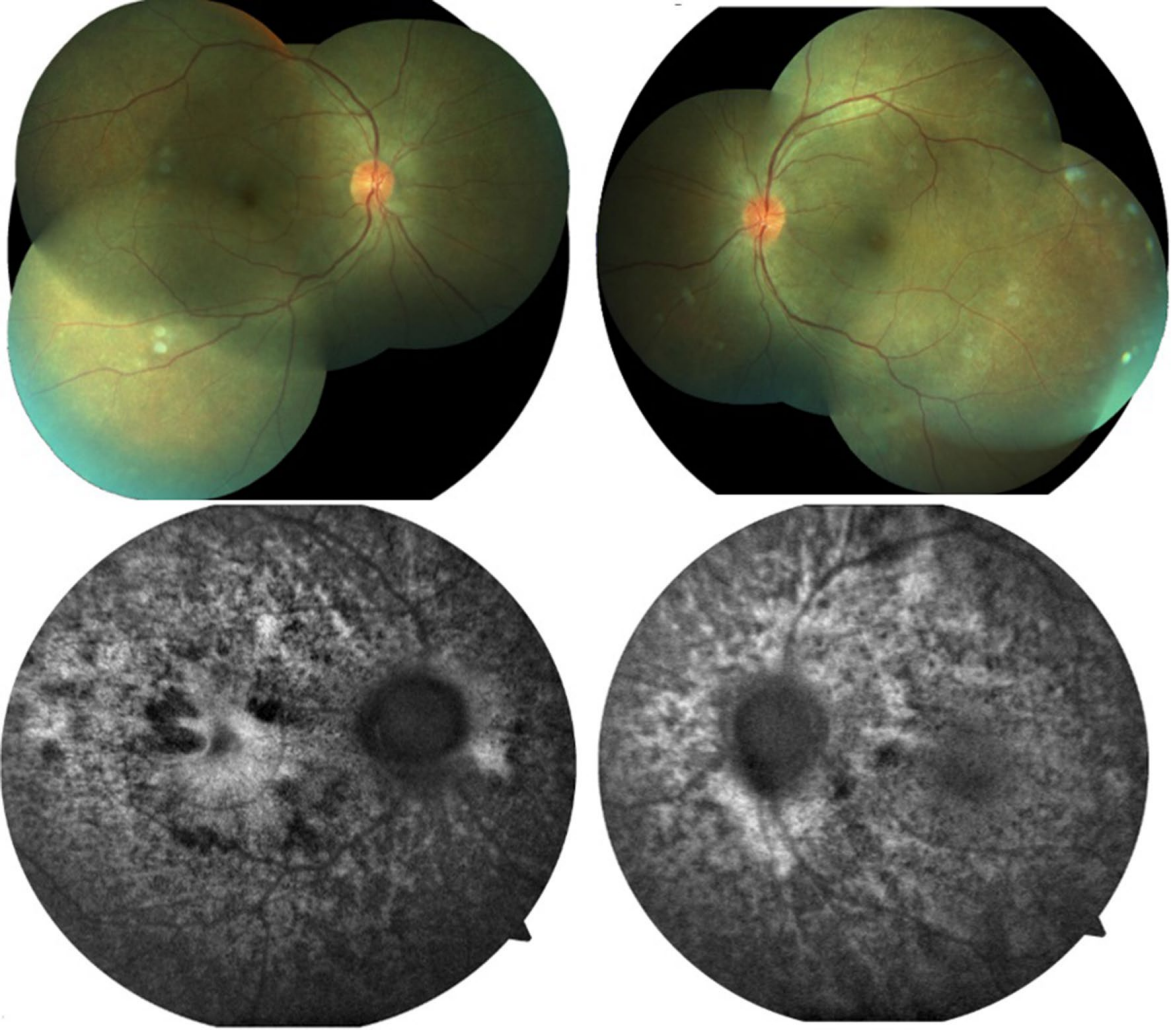
(Patient 2) Fundoscopy and autofluorescence of both eyes showing improvement of the serous retinal detachment

**Fig. 9:**
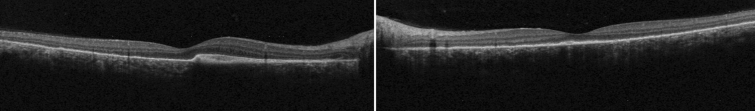
(Patient 2) OCT of both eyes showing resolution of the serous retinal detachment and bacillary layer detachment

Patient 2 was also treated with oral systemic prednisone (1 mg/kg/day, dose was taped by 10 mg every week). On the thirtieth treatment day her visual acuity achieved 20/25 OD and 20/50 OS, no signs of inflammatory activity and improvement of retinal detachment (Figs. 8 and 9).

Both patients have been followed up for the past 2 and 3 months respectively and have not had recurrence of the disease. Even with the COVID pandemic, these patients are being closely monitored due to the severity of the disease, with potential visual impairment.

## Discussion

Vogt-Koyanagi-Harada is a bilateral, autoimmune diffuse granulomatous uveitis associated with neurological, audiovestibular, and dermatological abnormalities. Although the exact pathogenesis is still uncertain, immunological and histopathological studies suggest that VKH is mediated by CD4+ T cells that target melanocytes. These activated T cells likely initiate the inflammatory process through generation of cytokines, IL-17 and IL23. Genetic factors and viral infections are likely involved [1–8].

Viral diseases may play a role in VKH development, as CMV seroprevalence is higher in VKH patients, and cross-reaction between tyrosinase peptides and cytomegalovirus antigen by T cells from patients with VKH has already been stablished [8]. Other virus, as Influenza A, was described as a trigger to VHK in a positive for HLA-DR4 patient [9]. Wade and cols. described a case of a 14-year-old female, without evidence of active *M. pneumoniae* infection, presented with elevated antibody titers and signs of VKH disease [19].

Bilateral uveitis with extraocular changes that were virtually identical to VKH was described in three patients with Hepatitis C (HCV). Two of them manifested the uveitis after the initiation of pegylated interferon–2b treatment. It suggests a possible association between the HCV infection and/or treatment with interferon and the development of VKH. The patients improved after corticosteroids and immunosuppressive treatment and suspension of the antiviral therapy [10, 11].

VKH can also associate with systemic autoimmune diseases, such as autoimmune polyglandular syndrome, Guillain-Barré syndrome, and immunoglobulin A nephropathy [20, 21].

Dogan et al. described a case of VKH following BCG vaccination in a patient treating superficial transitional cell carcinoma (TCC) of the bladder, and another one with both VKH and tuberculosis. The authors speculated that *M. tuberculosis* and BCG proteins induce high Th1 responses causing uveitis by antigenic mimicry [13].

Reports described a case of VKH following influenza, hepatitis B and yellow fever vaccination. One proposed mechanism is related to the use of adjuvants in vaccines. Adjuvants enhance immunogenic activity by a combination of mechanisms, including cytokine and chemokine release, sustained release of antigen (depot effect), activation of antigen presenting cells, antibody production, and cellular recruitment. Adjuvants are also routinely used in experimental auto-immune uveitis models [9–15].

The Oxford-AstraZeneca ChAdOx1 nCoV-19 vaccine (AZD1222) consists in a replication-deficient adenoviral vector, containing the SARS-CoV-2 structural surface glycoprotein antigen gene, and there are no adjuvants used in its composition. This vaccine induces generation of binding and neutralizing antibodies, and interferon-γ enzyme-linked immunospot responses [22]. Patel et al. describes a case of a 37-year-old man who was diagnosed with Guillain–Barre syndrome (GBS) 3 weeks post the first dose of the ChAdOx1 vaccine, in the absence of any other triggering factors. There are a few cases, which have been published correlating COVID-19 infection with the development of GBS [18].

Recently a case of Citokine Release Syndrome that occurred 5 days after vaccination with BTN162b2 (tozinameran)—the Pfizer-BioNTech mRNA COVID-19 vaccine—in a patient with colorectal cancer was reported [23].

Although it is difficult to determine causality, our cases raise the possibility of ChAdOx1 nCoV-19 (AZD1222) vaccination and COVID-19 triggering—or even causing—VKH disease. This report of vaccine-induced VKH can enlighten possible causative mechanisms involved in VKH pathogenesis. Sir. Stewart Duke-Elder, in 1966, had already emphasized that the etiology of numerous obscure uveitis may be secondary to virus infections [24]. The possibility of identifying which Sars-CoV-2 viral particles—possible used in the vaccines, as the structural surface glycoprotein antigen—may trigger uveitis is an important path for scientific research.

## Data Availability

All data generated during this study are included in this published article.
